# Different ways to die in a changing world: Consequences of climate change for tree species performance and survival through an ecophysiological perspective

**DOI:** 10.1002/ece3.5663

**Published:** 2019-10-02

**Authors:** Paulo Eduardo Menezes‐Silva, Lucas Loram‐Lourenço, Rauander Douglas Ferreira Barros Alves, Letícia Ferreira Sousa, Sabrina Emanuella da Silva Almeida, Fernanda Santos Farnese

**Affiliations:** ^1^ Laboratory of Plant Ecophysiology Instituto Federal Goiano – Campus Rio Verde Goiás Brazil

**Keywords:** climate change, drought, flooding, global warming, high CO_2_ concentration, tree mortality

## Abstract

Anthropogenic activities such as uncontrolled deforestation and increasing greenhouse gas emissions are responsible for triggering a series of environmental imbalances that affect the Earth's complex climate dynamics. As a consequence of these changes, several climate models forecast an intensification of extreme weather events over the upcoming decades, including heat waves and increasingly severe drought and flood episodes. The occurrence of such extreme weather will prompt profound changes in several plant communities, resulting in massive forest dieback events that can trigger a massive loss of biodiversity in several biomes worldwide. Despite the gravity of the situation, our knowledge regarding how extreme weather events can undermine the performance, survival, and distribution of forest species remains very fragmented. Therefore, the present review aimed to provide a broad and integrated perspective of the main biochemical, physiological, and morpho‐anatomical disorders that may compromise the performance and survival of forest species exposed to climate change factors, particularly drought, flooding, and global warming. In addition, we also discuss the controversial effects of high CO_2_ concentrations in enhancing plant growth and reducing the deleterious effects of some extreme climatic events. We conclude with a discussion about the possible effects that the factors associated with the climate change might have on species distribution and forest composition.

## Graphical abstract

The occurrence of extreme weather events will prompt profound changes in several plant communities, resulting in massive forest dieback events that can trigger a massive loss of biodiversity in several biomes worldwide. Despite the gravity of the situation, our knowledge regarding how extreme weather events can undermine the performance, survival, and distribution of forest species remains very fragmented. Therefore, the present review aimed to provide a broad and integrated perspective of the main biochemical, physiological, and morpho‐anatomical disorders that may compromise the performance and survival of forest species exposed to climate change factors, particularly drought, flooding, and global warming.

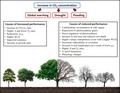

## 1 INTRODUCTION

Over the last decades, anthropogenic activities such as uncontrolled deforestation and increasing greenhouse gas emissions have resulted in a series of environmental imbalances that have caused significant changes in complex climate dynamics around the world. Examples of these changes include increases in the atmospheric CO_2_ concentration [CO_2_] as well as the global average temperature (IPCC, 2014). In addition to the changes already observed, several climate models predict an intensification of these factors in the ensuing decades, which may result in a higher incidence of extreme weather events (Zhang et al., 2015). According to these models, drastic reductions in rainfall are expected for some regions, whereas greater rainfall volumes are expected for others, resulting in more frequent and intense drought and flood events, respectively (IPCC, 2014). In addition to the changes in pluviosity patterns, alterations in thermic regimes are also expected in several regions worldwide, which may increase the intensity and frequency of extreme temperature events, such as heat waves and late‐spring frost events (IPCC, 2014).

Temperature and water availability are two of the main determinants of the establishment, distribution, and survival of plant species around the world (Anderegg & Hillerislambers, 2016; Bond, Woodward, & Midgley, 2005; Lu et al., 2017; Osland et al., 2017; Trueba et al., 2017). Thus, if climate model predictions are confirmed, the higher incidence, duration, and intensity of extreme weather can prompt profound changes in several plant communities, resulting in massive forest dieback, which can culminate in massive losses in biodiversity (Choat et al., 2012; Wiens, 2016). In fact, some studies report that climate change can trigger the replacement of several species from some biomes by those that are better adapted to extreme weather events. In tropical regions, for example, the occurrence of extreme weather events can lead to the replacement of the Amazon forest by Savannah vegetation (Lapola, 2007). A similar scenario of changes in species composition is also expected for several other vegetation types, such as grasslands, temperate, boreal, and Mediterranean forests (Barros, Thuiller, & Münkemüller, 2018; Boulanger et al., 2017; Brusca et al., 2013; Lucas‐Borja, 2016; Zhang, Niinemets, Sheffield, & Lichstein, 2018). However, it is important to emphasize that despite the fact that these extreme weather events are expected to intensify in the upcoming decades, their deleterious effects are already being observed. Indeed, a growing body of evidence associates the increasing death of forest species with water (drought and flood) and heat stresses (Allen et al., 2010; Anderegg, Flint, et al., 2015; Anderegg, Hicke, et al., 2015; Anderegg, Kane, & Anderegg, 2012; Choat et al., 2012; Greenwood et al., 2017; Liang, Leuschner, Dulamsuren, Wagner, & Hauck, 2016; Park Williams et al., 2012; Will, Wilson, Zou, & Hennessey, 2013). Observations of these widespread mortality events associated with extreme weather conditions have been documented on plants from all functional types, from all biomes around the world (McDowell et al., 2008). These observations become even more alarming when we take into account the proportion of plant species that may become extinct as a result of the intensification of such stresses (Becklin et al., 2016; Urban, 2015; Wiens, 2016).

In addition to the massive loss of biodiversity, the increase in forest death events can also promote profound changes in global carbon and water cycles (Frank et al., 2015). Indeed, as forests cover significant portions of the Earth's surface, and because they contribute to a large portion of primary carbon sequestration and water cycling, even minor environmental changes can disturb the complex dynamics of the terrestrial biosphere (Yan, Zhong, & Shangguan, 2017). Examples of such disturbances can be seen in recent studies that point to the widespread reduction in carbon sequestration in several regions of the globe, both in tropical and in temperate ecosystems (Doughty et al., 2015; Gatti et al., 2014; Greenwood et al., 2017; Taylor et al., 2017).

The great disturbances promoted by factors associated with climate change in plant communities reflect their impact on essentially all levels of plant organization. In fact, over the last years, numerous studies have demonstrated the diversity of strategies and defense mechanisms of plants exposed to extreme weather events, as well as the factors related to tree mortality under those conditions (Allen et al., 2010; Anderegg, Flint, et al., 2015; Anderegg, Hicke, et al., 2015; Bennett, McDowell, Allen, & Anderson‐Teixeira, 2015; Choat et al., 2012; Liang et al., 2016; McDowell et al., 2015; Mitchell et al., 2013; Park Williams et al., 2012; Rodríguez‐Calcerrada et al., 2017; Tognetti & Palombo, 2013). However, although our understanding of the mechanisms behind forest dieback has grown considerably, the knowledge about this topic still remains very fragmented. Therefore, the present review aimed to provide a broad and integrated perspective of the main biochemical, physiological, and morpho‐anatomical disorders associated with exposure to the stress factors triggered by extreme climatic events (mainly drought, flooding, and global warming), which may compromise the performance and, ultimately, the survival of tree species and forest ecosystems in a global scale. In addition, we also discuss the controversial effects of high [CO_2_] in enhancing plant growth and reducing the deleterious effects of some extreme climatic events. We conclude with a discussion about the possible effects that the factors associated with the climate change might have on species distribution and forest composition.

## 2 MAIN DRIVERS OF FOREST DIEBACK UNDER A SCENARIO OF CLIMATE CHANGE AND THEIR IMPACT ON KEY BIOCHEMICAL, PHYSIOLOGICAL, AND MORPHO‐ANATOMICAL ASPECTS

### 2.1 Drought

One of the main factors commonly associated with forest dieback events under drought conditions is the carbon starvation (CS) due to the depletion of nonstructural carbohydrates (NSCs), as a result of a negative carbon balance (Flexas, Bota, Galmés, Medrano, & Ribas‐carbo, 2006; McDowell et al., 2008; Mitchell et al., 2013; O'Grady, Mitchell, Pinkard, & Tissue, 2013; Weber et al., 2018). This imbalance involves a complex network of interconnected factors, which include the reduction in carbon assimilation via impairment of photosynthesis rates, both due to diffusive (e.g., reduced stomatal and mesophyll conductance) and biochemical (inhibition of specific metabolic processes) limitations (Flexas et al., 2009), and the increase in metabolic costs to repair disrupted structures (e.g., membranes, proteins, and nucleic acids), and to produce defense molecules, coupled with the possible increase in respiration (*R*) and photorespiration rates (*P*_R_; Dias & Brüggemann, 2010; Maroco, Rodrigues, Lopes, & Chaves, 2002; PARRY, 2002; Tezara, Mitchell, Driscoll, & Lawlor, 1999). However, although CS has been suggested as one of the main triggers of tree mortality under drought conditions (McDowell et al., 2008), this topic is still under intense debate due to some uncertainties. One of this uncertainties involves the fact that the vast majority of studies on C dynamics under drought conditions use only small tree segments (e.g., roots and shoots), which make it difficult to address the role of carbon storage and remobilization in whole‐tree dieback events (Hartmann et al., 2018; Kono et al., 2018). In addition, under severe stress conditions, plants also can use alternative molecules (e.g., lipids and organic acids) to fulfill the respiratory process (Araújo, Tohge, Ishizaki, Leaver, & Fernie, 2011; Pires et al., 2016; Weber et al., 2018), a strategy that is usually neglected in drought mortality studies. Thus, to better understand the impact that the fluctuations in the C metabolism may lead to the behavior of tree species under severe drought events, it is essential to deeply investigate the whole‐tree C dynamics and how the use of alternative energetic molecules can postpone the collapse of the respiratory metabolism (Hartmann et al., 2018). However, although the role of CS in triggering tree mortality remains controversial (Hartmann et al., 2018; Körner, 2015), the negative carbon balance induced by drought can have a profound impact on the dynamics of carbon flux on a global scale. In fact, some studies have associated drought events with a reduction in the primary productivity of several plant communities (Brzostek et al., 2014; Hilker et al., 2014; Yuan et al., 2016; Zhao & Running, 2010).

**Figure 1:**
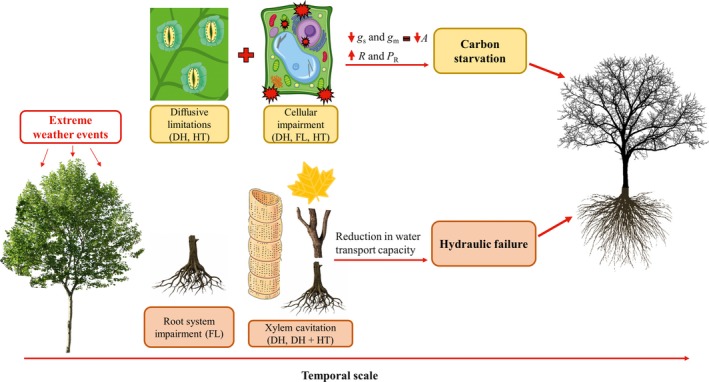
Main physiological disorders induced by drought (DH), flooding (FL), and heat stress (HT) which may reduce plant performance and survival under a climate change scenario. Overall, tree dieback events are mainly related to impairments in water transport and/or carbon balance. Reductions in water transport capacity are associated with hydraulic failure (as a consequence of xylem cavitation) and damages to the root system (reduced expression and activity of aquaporins as well as alterations in root morphology and growth). Conversely, negative carbon balance can be triggered by reductions in photosynthesis (*A*), as a result of diffusive (lower stomatal (*g*_s_) and mesophyll (*g*_m_) conductance) and biochemical (damages to membranes and enzymes) limitations, in addition to increases in respiration (*R*) and photorespiration (*P*_R_) rates

In addition to perturbations in the carbon balance, impaired water transport has also been suggested as a major factor causing death in plants exposed to drought (Anderegg et al., 2016; Choat et al., 2012; Corlett, 2016; McDowell et al., 2008; O'Brien et al., 2017; Rodríguez‐Calcerrada et al., 2017; Tai, Mackay, Anderegg, Sperry, & Brooks, 2016). This occurs because despite the partial closure of stomata, decreased water availability can considerably increase the tension in xylem vessels, a process that may result in cavitation (Tyree & Sperry, 1989). As a consequence, cavitation can lead to extensive hydraulic failure (HF), reducing a plant's ability to replenish the water lost through transpiration and resulting in extreme desiccation and death (Mitchell et al., 2013; Rodríguez‐Calcerrada et al., 2017; Rowland et al., 2015) (Figure 1a). In fact, several studies have emphasized the HF as the main determinant of the drought‐induced tree mortality across contrasting vegetation types (e.g., tropical, temperate, boreal, and Mediterranean forests), whereby species less vulnerable to cavitation tend to be more drought‐tolerant (Anderegg, Anderegg, Kerr, & Trugman, 2019; Anderegg, Flint, et al., 2015; Choat et al., 2012; Greenwood et al., 2017; O'Brien et al., 2017). Vulnerability to cavitation, in turn, is directly related to a series of anatomical (pit membranes, diameter, and frequency of xylem vessels), morphological (wood density, root depth, sapwood to leaf area), and physiological (control of stomatal movement and phenological stage) factors, which vary widely between species and functional groups (Greenwood et al., 2017; Jacobsen, Ewers, Pratt, Paddock, & Davis, 2005; Lens et al., 2011; Markesteijn, Poorter, Paz, Sack, & Bongers, 2011; McAdam & Brodribb, 2016; Santiago et al., 2004; Scoffoni et al., 2017; Trueba et al., 2017). Despite this great variability in vulnerability to cavitation, recent studies have shown that most forest communities around the globe operate within a very narrow hydraulic safety margin, placing species from virtually all biomes at risk (Choat et al., 2012). However, although HF has been pointed as a major determinant of tree mortality (Anderegg, Flint, et al., 2015; Hartmann et al., 2018), the lack of key information regarding the stability of water transport under drought makes it difficult to predict the behavior of trees under such conditions. For example, until now there is no consensus about what is the lethal point in which xylem embolism develops into HF (Hartmann et al., 2018), especially in angiosperms, and neither if some mechanisms of xylem repair, as the controversial refiling (Charrier et al., 2016; Sperry, 2013), can reestablish the water transport after extreme drought events. Thus, it is of pivotal importance to determine specific thresholds of recovery and fatal embolism and to better understand whether the mechanisms of xylem repair can mitigate the occurrence of such catastrophic events (Hartmann et al., 2018).

Another uncertainty regarding the role of HF in drought‐induced tree mortality is the fact that most of the studies that seek to investigate the vulnerability to cavitation in trees are made in leaves and segments of branches (e.g., trunks and roots). Thus, little is known about in which scale other more basal organs are affected by extreme drought events (Mcculloh, Johnson, Meinzer, & Woodruff, 2014). This is a matter of great relevance, especially when we take into account the hypothesis of hydraulic segmentation, which postulates that more distal organs (leaves and small branches), which represent a lower carbon investment, tend to be more vulnerable to cavitation than more basal organs (trunk and roots) (Charrier et al., 2016; Choat, Lahr, Melcher, Zwieniecki, & Michele, 2005; Tyree & Zimmermann, 2002). In this sense, to better understand the real impact of drought on water transport in different species, it is essential to better characterize the events of cavitation in different organs. This type of integrative approach has become possible thanks to the emergence of noninvasive methodologies such as the “optical vulnerability technique (OV)” and the “X‐ray computed microtomography (micro‐CT),” which allow the in vivo monitoring of the emergence and propagation of embolism events in different organs of plants exposed to drought (Brodribb et al., 2016; Charrier et al., 2016; Choat et al., 2016 ; Rodriguez‐Dominguez, Carins Murphy, Lucani, & Brodribb, 2018).

In addition to a higher tolerance against cavitation, the efficient control of water loss also represents a central component of the tree survival under drought. In fact, a recent study has shown that most of the species tend to close their stomata before the onset of the cavitation events (Martin‐StPaul, Delzon, & Cochard, 2017). Nevertheless, even after the complete stomatal closure, the plants keep losing water to the atmosphere through their cuticle (Bueno et al., 2019). This residual transpiration (*g*_min_) varies widely across species and functional groups (Schuster, Burghardt, & Riederer, 2017), and can directly affect the time to HF (Cochard, 2019). However, although *g*_min_ has been recently pointed as a central component of the drought tolerance strategy (Cochard, 2019), several questions regarding this trait remain. For example, what are the main determinants of the variability of *g*_min_ across species? Is there a coordination between *g*_min_ and vulnerability to cavitation? To which extent do plants are able to adjust this trait through drought acclimation? The lack of consensus regarding the answers to these questions highlights an important gap in our understanding of the strategies of water conservation, both within and across tree species. Thus, in order to better predict the impact of drought on forest communities, it is essential to unravel the uncertainties about *g*_min_ and also to widespread the integration of this trait on climate change models.

Although tree mortality under drought conditions can be triggered by a combination of different effects (Adams et al., 2017; Hartmann et al., 2018; Kono et al., 2018), the impact of such effects can vary significantly depending on the phenological stage and size of the plant (Liu et al., 2019; Olson et al., 2018). As trees grow taller, the distance to move water from roots to leaves gets longer, which increases the resistance along the pathway (Ambrose, Sillett, & Dawson, 2009; Fajardo, McIntire, & Olson, 2019), resulting in an increase in the tension inside xylem vessels and in the risk of HF under drought conditions (Bennett et al., 2015; Fajardo et al., 2019). In fact, several studies already showed that taller trees, both within and across species, are more prone to drought‐induced cavitation, which greatly explain the reduction in growth, and the higher incidence of branch and whole dieback of taller individuals worldwide (Bennett et al., 2015; Fajardo et al., 2019; Lindenmayer & Laurance, 2016). To keep the water transport and avoid the risks of HF, taller trees usually display a set of morpho‐physiological alterations on their hydraulic system, such as the reduction in leaf area to sapwood area (Ambrose et al., 2009; McDowell et al., 2002). Although these alterations can significantly compensate for the reduction in hydraulic efficiency, the reduced leaf area can potentially limit the global carbon assimilation of taller trees, possibly making them more prone to carbon starvation on extreme drought events (Liu et al., 2018). Conversely, other studies have shown that the set of adjustments in the hydraulic system to deal with the increased stature might increase the hydraulic safety margin and, consequently, the resilience of taller trees in a scenario of reduced precipitation (Ambrose et al., 2009). In fact, a recent study showed that taller Amazonian forests are less sensitive to precipitation variation (Giardina et al., 2018), a result that contrast directly with other studies which show that larger trees suffer more during drought events in forests worldwide (Bennett et al., 2015; Olson et al., 2018). These contrasting results highlight the uncertainties about how species with different ages and heights will respond to the increase in drought intensity and duration. In order to shed light on this controversial topic, there is an urgent need to better understand how the differences in water uptake (e.g., root density and depth) and storage capacity (e.g., trunk capacitance) between small and taller plants can impact the stability of water transport of those trees under drought conditions (Hartmann et al., 2018; Martinez‐Vilalta, Anderegg, Sapes, & Sala, 2019).

**Figure 2:**
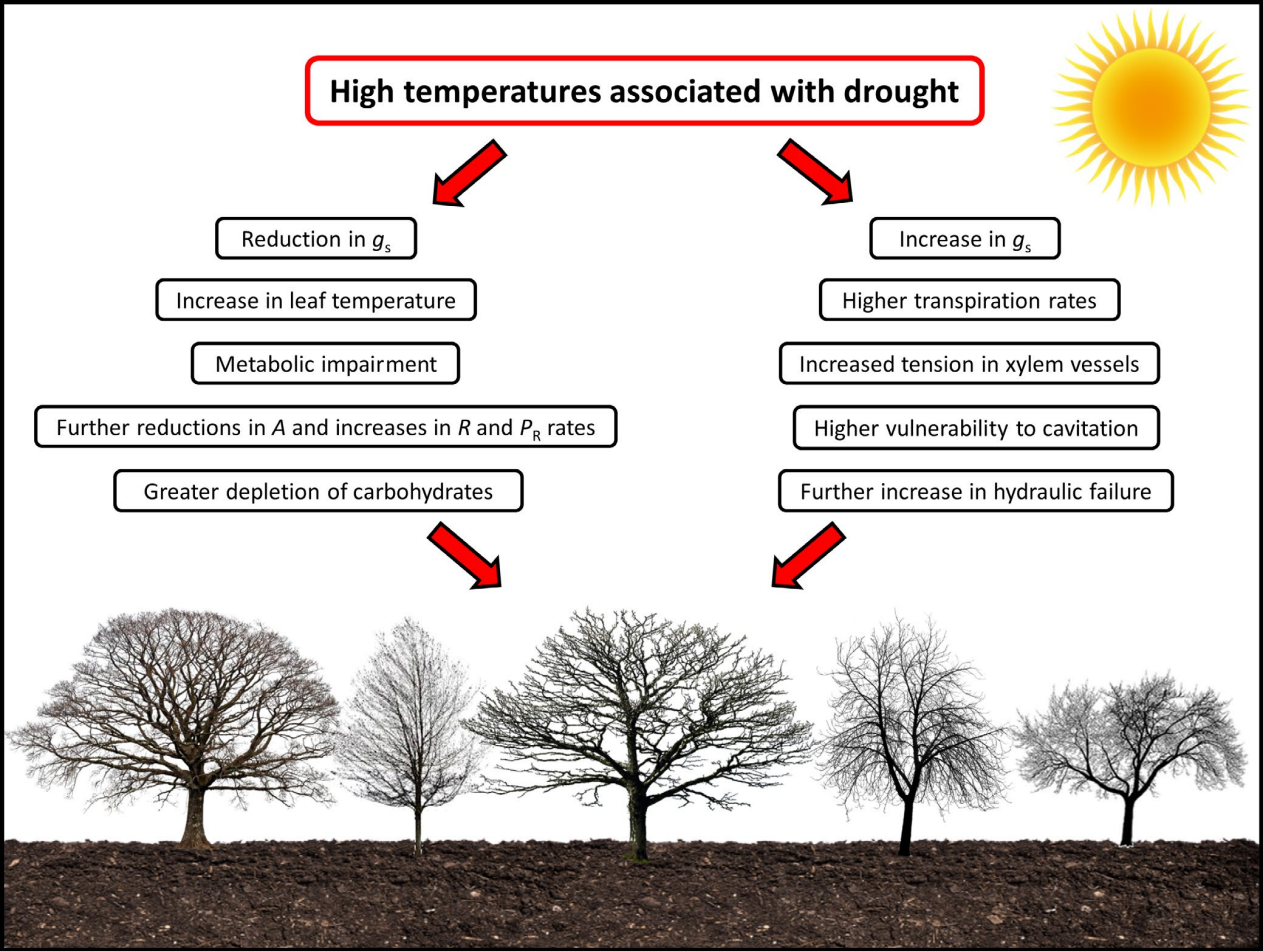
Global warming increases the vulnerability of tree species under drought conditions in different ways. For some species, high temperatures induce the reduction in stomatal conductance (*g*_s_), reducing leaf transpiration and increasing leaf temperature, which may lead to deeper reductions in photosynthesis (*A*) and increases in photorespiration (*P*_R_) and respiration (*R*) rates, further intensifying the negative carbon balance induced by drought. For other species, high temperatures increase the stomatal conductance and leaf transpiration, further increasing the tension in xylem vessels induced by drought, leading to higher vulnerability to cavitation and hydraulic failure

The factors that drive forest dieback also appear to vary significantly according to the intensity and duration of the drought event. Plants exposed to moderate but prolonged drought may not reach the critical water potentials that induce HF but may experience a lethal reduction in carbohydrate levels due to the negative carbon balance (McDowell et al., 2008, 2011; Mitchell et al., 2013; O'Brien, Leuzinger, Philipson, Tay, & Hector, 2014). Nonetheless, brief periods of severe drought can result in the inability to regulate water status and can induce death by HF (Anderegg et al., 2016; Choat et al., 2012; Delzon & Cochard, 2014; Mitchell et al., 2013; Rodríguez‐Calcerrada et al., 2017; Rowland et al., 2015) (Figure 1a). Thus, the extent of damage caused by drought, as well as the time required to induce plant death under these conditions, can vary significantly according to the growth and water management strategies of a given species (e.g., isohydric or anisohydric species; Allen et al., 2010; Mitchell et al., 2013; Reyer et al., 2013). It should be noted that under both of the above scenarios, the deleterious effects of drought and the possible occurrence of forest dieback can be intensified by the combined action of high temperatures (Allen, Breshears, & McDowell, 2015; Dai, 2012; Park Williams et al., 2012) (Figure 2). As an example, some mechanisms used to minimize water loss under drought conditions (e.g., reductions in *g*_s_) tend to decrease leaf cooling via transpiration, a process that can induce a series of cell injuries (discussed in the next sections), and increase respiratory and photorespiratory activities, further disturbing the carbon balance (Flexas et al., 2006; Mitchell et al., 2013) (Figure 2). Conversely, for other species, the increased temperature may also enhance plant transpiration, thus increasing the vulnerability to cavitation under drought conditions (Park Williams et al., 2012; Will et al., 2013) (Figure 2).

In addition to intensity and duration, the frequency of drought events appears to be a predominant factor in forest dieback events, yet it is rarely taken into account in climate models. In fact, few studies to date have focused on the behavior of plants subjected to cyclical drought episodes, even though this is a more common situation than isolated events (Menezes‐Silva et al., 2017). Recent studies have also shown that after exposure to severe drought, years may be required for several plant communities to fully recover their physiological processes (e.g., growth rates), making them even more vulnerable to further drought episodes (Anderegg, Schwalm, et al., 2015). Evidence of this reduced ability to fully recover after severe drought events can be observed in plants from contrasting vegetation types, such as the temperate and continental forests (Gazol et al., 2018). Thus, an increase in the frequency of drought events can significantly impair the composition of several biomes around the world. Therefore, in addition to a better understanding of the factors associated with reduced performance under drought conditions, it is vital to increase our knowledge about how plants recover from these extreme climatic events.

Finally, another factor that can increase the vulnerability of forest ecosystems in a scenario of reduced precipitation is the increase in the frequency and intensity of fire events. In fact, due to the increase in litter production and reduction in biomass humidity (Collins, Bennett, Leonard, & Penman, 2019; Duursma et al., 2016), the effect of drought on vegetation can significantly increase the fire spread and intensity, even in those regions that would be unlike to be burned (e.g., mesic sites and poleward‐facing slopes; Collins et al., 2019; Krawchuk et al., 2016; Leonard, Bennett, & Clarke, 2014). It is important to note that although plants may have a set of traits and strategies to allow them to survive fire episodes and/or recompose burned areas (Hoffmann et al., 2009; Pellegrini, Franco, & Hoffmann, 2016; Schafer, Breslow, Hohmann, & Hoffmann, 2015), the increase in drought intensity can significantly reduce the resilience of tree species from fire‐prone habitats (Pratt, Jacobsen, Ramirez, & Helms, 2014). Some studies already showed that the negative impact of drought on seed germination and seedling survival can drastically reduce the postfire regeneration of subalpine species (Harvey, Donato, & Turner, 2016), which may lead to a shift toward more drought‐tolerant species (Moser, Temperli, Schneiter, & Wohlgemuth, 2010). In addition to the negative impact on seedling recruitment, drought can also affect the regeneration of burned areas thought the increase in the vulnerability of resprouting plants (Pratt et al., 2014). In fact, although it was already shown that resprouting species often display a better water status in the months after the crown fire (probably due to reduced leaf area and thus higher root‐to‐shoot ratio; Clemente, Rego, & Correia, 2005; Ramirez, Pratt, Jacobsen, & Davis, 2012; Refsland & Fraterrigo, 2018; Schwilk, Brown, Lackey, & Willms, 2016), they tend to be more vulnerable to drought than co‐occurring unburned plants (Ramirez et al., 2012; Saruwatari & Davis, 1989). An example of the higher vulnerability of resprouting plants is the well‐documented increase in mortality rates of shrub species from a chaparral community when intense drought occurred in the following year after a fire event (Pratt et al., 2014). This increase in postfire mortality is commonly attributed to a reduction in cavitation resistance (Jacobsen, Tobin, Toschi, Percolla, & Pratt, 2016; Pratt et al., 2014) and probably is also linked to a depletion in carbohydrate reserves of the resprouting plants (McDowell et al., 2008). Thus, the increase in drought frequency, intensity, and duration has the potential to not only change the dynamics of fire regimes (Clarke, Knox, Bradstock, Munoz‐Robles, & Kumar, 2014; Littell, Peterson, Riley, Liu, & Luce, 2016), putting in risk fire‐sensitive species from regions which were unlike to be burned, but can also increase the vulnerability from fire‐tolerant species, resulting in drastic changes in forest composition and loss of biodiversity (Henzler, Weise, Enright, Zander, & Tietjen, 2018).

### 2.2 Flooding

Under flooding conditions, the reduction in oxygen availability can induce a number of physiological imbalances that strongly impact key aspects of the growth, development, and survival of flooded species (Guo, Huang, Xu, & Zhang, 2011; Li et al., 2015). The susceptibility, extent of damage, and lifespan of flooded plants vary widely among species and depend on the ability to invoke a series of morpho‐anatomical (production of lenticels, adventitious roots, and aerenchyma), biochemical (increased fermentative metabolism), and physiological (increased ethylene production) adjustments (Bailey‐Serres & Colmer, 2014; Herrera, 2013; Voesenek & Bailey‐Serres, 2015). Common effects of exposure to flooding stress, especially for sensitive species, include inhibition of root and shoot growth, leaf necrosis, bark damage, increased ROS production, and several other metabolic disorders (Ferner, Rennenberg, & Kreuzwieser, 2012; Gupta & Igamberdiev, 2016; Kreuzwieser & Rennenberg, 2014; Liu, Cheng, Xiao, Guo, & Wang, 2014; Steffens & Rasmussen, 2016; Voesenek & Bailey‐Serres, 2015). Despite such general damage, evidence shows that the main factor related to the death of flooded plants involves carbon balance disruption, specifically changes in photosynthetic and respiratory processes (Li et al., 2015) (Figure 1b).

Reductions in *A* rates of flooded plants have been widely documented (Kreuzwieser & Rennenberg, 2014; Li et al., 2015; Liu et al., 2014; Martínez‐Alcántara et al., 2012), especially for sensitive species (Argus, Colmer, & Grierson, 2015), and appear to be primarily related to both stomatal and nonstomatal limitations (Kreuzwieser & Rennenberg, 2014). The latter include reduced concentrations of photosynthetic pigments (Ojeda, Schaffer, & Davies, 2004), decreased Rubisco content and activity (Herrera, 2013), and accumulation of soluble sugar in leaves, which may induce negative feedback on photosynthesis (Ferner et al., 2012; Kreuzwieser & Rennenberg, 2014). In contrast, stomatal limitations are largely associated with reductions in root hydraulic conductivity (Else, Coupland, Dutton, & Jackson, 2001; Else, Davies, Malone, & Jackson, 1995; Islam & Macdonald, 2004; Li et al., 2015; Zhang & Davies, 1986) (Figure 1b). The occurrence and extent of these hydraulic limitations appear to depend on the combination of damage, lower growth rates, and suberization of the root system, in association with lower expression and abundance of aquaporins (Islam & Macdonald, 2004; Kreuzwieser & Rennenberg, 2014; Li et al., 2015). As a result, decreased root hydraulic conductivity can limit water and nutrient absorption and, paradoxically, induce shoot desiccation, which reduces leaf water potential and, consequently, *g*_s_ (Kreuzwieser & Rennenberg, 2014; Li et al., 2015). In addition to hydraulic components, the transport of signaling molecules from roots to leaves [e.g., abscisic acid (ABA)] and changes in the pH of phloem sap also appear to be related to reductions in *g*_s_ in flooded plants (Herrera, 2013; Kreuzwieser & Rennenberg, 2014; Li et al., 2015).

Alterations in respiratory metabolism are equally important in species death under flooding conditions (Ferner et al., 2012; Kreuzwieser & Rennenberg, 2014; Martínez‐Alcántara et al., 2012). These changes are largely due to the switch from aerobic to fermentative metabolism induced by the low concentration of oxygen in flooded soils (Bailey‐Serres, Lee, & Brinton, 2012; Kreuzwieser et al., 2009; Kreuzwieser & Rennenberg, 2014; Loreti, Veen, & Perata, 2016). As fermentation generates approximately 16 times less energy than oxidative phosphorylation, an energy deficit is expected under flooding conditions, particularly if photosynthesis is also reduced. This energy imbalance can be partially overcome through consumption of reserve materials (e.g., nonstructural carbohydrates and lipids) and/or decreases in the activity of energy‐intensive processes (e.g., nitrogen (N) assimilation and cell wall formation; Christianson, Llewellyn, Dennis, & Wilson, 2010; Kolb, Rawyler, & Braendle, 2002; Kreuzwieser et al., 2009; Le Provost et al., 2012; Loreti, Valeri, Novi, & Perata^2018^). However, depending on the flooding duration, reserve consumption can lead to carbon deprivation, making the plant even more vulnerable to this and other stresses (e.g., high temperatures and pathogen attack). Moreover, decreased reserve levels may also limit and/or increase the time required for a plant to fully recover its physiological activities (Li et al., 2015; Loreti et al., 2016), placing species inhabiting regions that undergo recurrent flooding events at even greater risk (Angelov et al., 1996). Thus, the ability to maintain a positive carbon balance appears to be one of the main determinants of the survival of species in flooding situations (Li et al., 2015; Loreti et al., 2018), even more so in a scenario that forecasts more frequent and intense flooding events (Lehmann, Coumou, & Frieler, 2015).

### 2.3 Global warming and heat stress

Plant responses to high temperature, in addition to the damage triggered by this stress, vary widely among species and functional groups (Klockmann, Günter, & Fischer, 2017; Marias, Meinzer, & Still, 2017; O'Sullivan et al., 2013; Slot & Winter, 2017; Teskey et al., 2015; Wujeska‐Klause, Bossinger, & Tausz, 2015). Accordingly, the susceptibility of a plant to extremely high temperatures, a situation commonly observed during heat waves, appears to depend on a series of characteristics and adjustments at morpho‐anatomical (crown architecture, leaf size, and shape), physiological (transpiration rate and maximum stomatal conductance), and molecular (production of heat shock proteins, low‐weight compounds, and activation of the antioxidative defense system) levels (Bita & Gerats, 2013; Galmés, Kapralov, Copolovici, Hermida‐Carrera, & Niinemets, 2015; Griffin & Prager, 2017; Obata et al., 2015; Scafaro et al., 2016; Slot & Winter, 2017; Teskey et al., 2015; Wujeska‐Klause et al., 2015; Zhang et al., 2005). Moreover, the vulnerability of a species to high temperatures also depends on its growth strategy. For example, in tropical forests, fast‐growing plants in high‐light environments tend to be more tolerant to high temperatures than slow‐growing species typical of shadier locations (Slot, Garcia, & Winter, 2016; Slot & Winter, 2017; Wright et al., 2004). Although there are many factors that may influence species susceptibility to high temperature, it is important to note that most of the forest dieback events across several biomes appear to involve the association of this stress with drought (Adams et al., 2009; Allen et al., 2015, 2010; Park Williams et al., 2012; Will et al., 2013) (Figure 2). Similarly, high temperature combined with flooding can further compromise the performance of plant species through additional damages to the photosynthetic process, changes in root respiration, and also by compromising the synthesis of structural components (e.g., cell wall). However, it is important to note that only a few studies have addressed the links between these two stresses in great detail (Chen et al., 2017a, 2017b; Donovan, Stumpff, & McLeod, 1989; Lin, Lin, Syu, Tang, & Lo, 2016), and thus, our knowledge about this topic, especially on wood species, is rather fragmented.

Among the physiological processes that are affected by high temperatures, photosynthesis has received the most attention (Drake et al., 2016; Hüve, Bichele, Rasulov, & Niinemets, 2011; Slot & Winter, 2017; Teskey et al., 2015; Urban et al., 2017). In general, an increase in temperature increases *A* rates up to an optimal point, above which the process begins to be inhibited and may even reach zero (Slot & Winter, 2017). This reduction in the photosynthetic process has commonly been attributed to disruption of the photosynthetic electron transport chain in association with increased fluidity of thylakoid membranes and/or damage to photosystem II (Griffin & Prager, 2017; Hüve et al., 2011; Sharkey, 2005; Slot & Winter, 2017; Yamori, Hikosaka, & Way, 2014) (Figure 1c). In addition to structural damage, exposure to high temperatures may also result in inactivation of the enzyme Rubisco activase, which may lead to reduced availability of active Rubisco and thereby in a reduction in CO_2_‐fixation capacity (Sage, Way, & Kubien, 2008; Salvucci, 2004; Scafaro et al., 2016).

Similar to the responses observed for photosynthesis, an increase in temperature also enhances *R* rates to an optimal point, above which cell damage hinders respiration (Griffin & Prager, 2017; O'Sullivan et al., 2013). The optimal temperature and thermal limit of respiration are significantly higher than those of photosynthesis (O'Sullivan et al., 2013; Teskey et al., 2015). Due to the different thermal sensitivities of these two metabolic processes, higher temperatures tend to increase the *R*/*A* ratio, resulting in a significant reduction in daily carbon fixation (Stangler, Hamann, Kahle, & Spiecker, 2017; Zhao, Hartmann, Trumbore, Ziegler, & Zhang, 2013). In addition to reducing the amount of CO_2_ fixed, exposure to high temperatures may also lead to a decrease in carbohydrate reserves (e.g., starch) in response to enhanced maintenance respiration (associated with protein turnover and membrane repair; Hüve et al., 2011) (Figure 1c). Carbon balance may also be negatively affected by changes in Rubisco specificity and by reductions in CO_2_ solubility relative to O_2_, a process that may increase *P*_R_ rates (Carmo‐Silva et al., 2012; Carmo‐Silva, Scales, Madgwick, & Parry, 2015; Galmés et al., 2015). Thus, even small increases in temperature can cause significant reductions in the net productivity of several plant communities, making these plants even more vulnerable to the effects of the stress factors discussed above (Adams et al., 2009; Liang et al., 2016; McDowell & Allen, 2015; Park Williams et al., 2012). Overall, it is clear that the increase in global mean temperature, combined with other stressors, will have a devastating effect on the productivity and composition of various plant communities and, as a result, may lead to profound changes in the global carbon cycle.

An indirect effect of global warming that may also impact the performance and survival of tree species, especially under drought conditions, is the increase in the vapor pressure deficit (VPD) between leaf and atmosphere (Slot & Winter, 2017; Will et al., 2013). In fact, the increased evapotranspiration demand induced by high VPD can impact plant physiological processes in different ways (Figure 2). For some species, high VPD may enhance water loss and increase the vulnerability to cavitation (Adams et al., 2009; Park Williams et al., 2012), while for others, this factor can trigger reductions in *g*_s_, limiting CO_2_ diffusion for photosynthesis and leaf cooling through transpiration, which may impact carbon balance (due to cell damages and increased *R* and *P*_R_ rates; Bauweraerts et al., 2013; Duursma et al., 2014; Flexas et al., 2006; McDowell et al., 2008; Teskey et al., 2015) (Figure 2). These contrasting responses also show that the dynamics of stomatal movements can be extremely variable under high temperature, with some studies reporting increases (Freeden & Sage, 1999; Mott & Peak, 2010; Schulze, Lange, Evenari, Kappen, & Buschbom, 1974; Urban et al., 2017), decreases (Slot & Winter, 2017), or no changes (Cerasoli et al., 2014; Sage & Sharkey, 1987; Teskey, Bongarten, Cregg, Dougherty, & Hennessey, 1987; Vargas & Cordero, 2013;) in *g*_s_ under those conditions. This wide range of responses is attributed not only to the different sensitivities of species to VPD, but also to water potential, and internal CO_2_ concentration, combined with other factors (e.g., wind and water availability; Addington, Mitchell, Oren, & Donovan, 2004; Ocheltree, Nippert, & Prasad, 2014; Schymanski, Or, & Zwieniecki, 2013; Teskey et al., 2015; Yan et al., 2017). However, despite this great variability of responses, it should be noted that high VPD, as a result of increased temperature, has been suggested as a primary driver of tree mortality in different regions worldwide (Anderegg et al., 2012; Park Williams et al., 2012; Will et al., 2013) (Figure 2).

The uncertainties regarding the alterations of the water‐saving strategies of plants exposed to high temperature go beyond the stomatal movements. In fact, the dynamic of *g*_min_ rates in a scenario of increased temperature also represents an important gap in our knowledge. For most species, especially the nondesert ones, *g*_min_ rates tend to show small variations at temperatures from 15 to 35°C, while temperatures above 35° induce a drastic increase in cuticle permeability and, consequently, in water loss (Schreiber, 2001; Schuster et al., 2017). This abrupt increase in *g*_min_ rates under a certain temperature threshold, also known as transition temperature (Schuster et al., 2016), can have a catastrophic effect on plants exposed to heat waves, especially under drought conditions, since the increase in water loss can significantly increase the tension in xylem vessels, which can result in a reduction in the time to HF (Cochard, 2019). However, despite the great impact that the increase in atmospheric temperature can have on *g*_min_ rates (Bueno et al., 2019; Schuster et al., 2016) and, thus, on plant survival (Cochard, 2019), key questions regarding this topic remain. For example, we currently do not have enough information to answer to which extent plants acclimated to high temperatures can alter the physicochemical properties of their cuticle in order to increase the transition temperature. This kind of information is essential to improve the prediction about the impact that the increase in atmospheric temperature, especially in association with drought, will have on the composition of forest ecosystems worldwide (Cochard, 2019).

The effects of global warming are expressed not only through the increase in the occurrence of extremely high temperatures (e.g., heat waves), but also with more subtle changes, as the increase in winter temperatures. In fact, in the last century, an expressive increase in mean winter temperatures was observed in some regions (e.g., northern Europe; Mikkonen et al., 2015). However, although the occurrence of extremely low temperatures is expected to decrease in the decades to come (IPCC, 2014), paradoxically, the global warming will probably increase the vulnerability of plant species to frost‐induced injury, especially those from temperate regions (Augspurger, 2013; Príncipe et al., 2017). The process of cold acclimation is triggered by the reduction in temperatures and photoperiod and involves a marvelous set of biochemical adjustments (e.g., accumulation of soluble sugars, hydrophilic proteins, antioxidants, and chaperones) that confers cryoprotection to the cells (Basler & Körner, 2012). In early spring, as the temperature rises, the resistance to frost injury decreases progressively, reaching a minimum when new leaves emerge, making the plants extremely vulnerable to a “late‐spring” frost event (Lenz, Hoch, Vitasse, & Körner, 2013; Vitasse, Lenz, Hoch, & Körner, 2014; Vitasse, Schneider, Rixen, Christen, & Rebetez, 2018). Some studies already showed that these frost damages are related to a previous warm period, which induces precocious spring phenology (Vitasse et al., 2018). In this way, the phenological changes induced by the combination of warmer springs and large temperature fluctuations, predicted for the decades to come (IPCC, 2014), may significantly increase the vulnerability to frost in several tree species (Augspurger, 2013; Julio Camarero, Gazol, Sancho‐Benages, & Sangüesa‐Barreda, 2015). The occurrence of frost‐induced mortality events, especially those related to late‐spring frost, is considerably growing in some regions and is expected to be more frequent as the atmospheric temperature keeps changing (Augspurger, 2013). Moreover, it is important to note that these events of frost mortality are observed not only in temperate regions, but also in tropical forests. In a recent study, it was shown that a widespread mortality event in a tropical dry forest from Mexico was related to an unusual combination of duration, intensity, and timing of a frost event (Bojórquez, Álvarez‐Yépiz, Búrquez, & Martínez‐Yrízar, 2019). Similarly, dieback events related to frost injury were also reported for species from the Mediterranean region (Jalili et al., 2010; Matusick, Ruthrof, Brouwers, & Hardy, 2014). These examples illustrate well the impact of global warming on the vegetation, since its effects, especially the large temperature fluctuations, can significantly increase the vulnerability of forest species in virtually all biomes.

## 3 THE CONTROVERSIAL ROLE OF CARBON DIOXIDE: A POTENTIAL FRIEND OR A CERTAIN ENEMY?

The growing increase in the atmospheric [CO_2_] is one of the main effects of anthropic activities. In fact, from the industrial revolution to the present, [CO_2_] has increased from approximately 280 to 400 ppm. If the current greenhouse gas emission pattern is maintained, [CO_2_] is expected to reach levels of 750–1,300 ppm by the end of this century (IPCC, 2014). This significant rise in [CO_2_] may place a large number of species at risk because the weather extremes previously discussed are directly related to the increase in the concentration of this gaseous molecule (Becklin, Walker, Way, & Ward, 2017; Warren, Jensen, Medlyn, Norby, & Tissue, 2015). However, although CO_2_ is considered one of the main villains of climate change, its real effect on the performance and survival of forest species remains extremely controversial (Ellsworth et al., 2017; Friedlingstein et al., 2014; Schimel, Stephens, & Fisher, 2015; Sitch et al., 2015). For example, in addition to the central role of CO_2_ in driving climate change, several studies have shown that higher [CO_2_] can enhance the performance and productivity of forest species (Drake et al., 2011; Lewis, Lloyd, Sitch, Mitchard, & Laurance, 2009; Norby et al., 2005; Norby, Wullschleger, Gunderson, Johnson, & Ceulemans, 1999; Yang, Donohue, Mcvicar, Roderick, & Beck, 2016), as well as mitigate the deleterious effects of some abiotic stresses (Oliveira, Silva, & Carvalho, 2016; Rodrigues et al., 2016; Roy et al., 2016; Swann, Hoffman, Koven, & Randerson, 2016).

**Figure 3:**
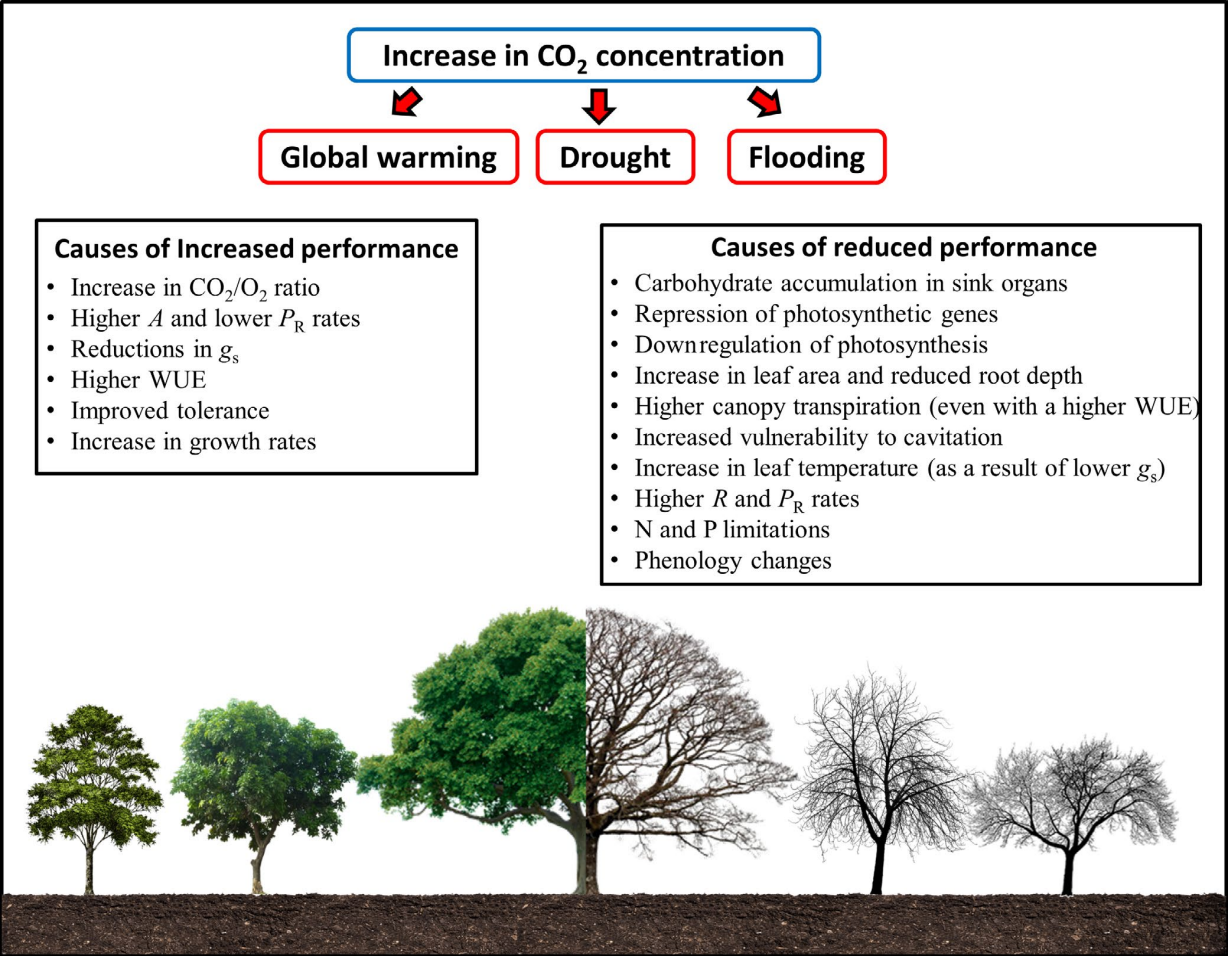
The controversial role of CO_2_. High [CO_2_] can stimulate plant growth through the increase in CO_2_/O_2_ ratio inside the chloroplasts, which enhance photosynthesis (*A*) and reduce photorespiration (*P*_R_), besides contributing to the increase in water use efficiency (WUE), as a result of reduced stomatal conductance (*g*_s_), thus minimizing the deleterious effect of some abiotic stresses. On the other hand, the fertilizer effect of high [CO_2_] is not always observed or only transitory, as a result of the downregulation of *A*, which is related to carbohydrate accumulation, repression of photosynthetic genes, and lower availability of nitrogen (N) and phosphorus (P). Moreover, some morphological and morpho‐anatomical changes induced by high [CO_2_] may also enhance the vulnerability of tree species to abiotic stresses, as a result of increased transpiration, reduced water uptake, increased vulnerability to cavitation, and higher respiration (*R*) and photorespiration (*P*_R_) rates. High [CO_2_] can also compromise the interactions between plants and their pollinators, through phenology changes

One of the most significant effects of the increase in [CO_2_] is the enhancement of the photosynthetic process, especially in C3 plants (Ainsworth & Long, 2005; Bader, Siegwolf, & Körner, 2010; Drake & Leadley, 1991; Faralli, Grove, Hare, Kettlewell, & Fiorani, 2017; Idso & Kimbal, 1997; Rey & Jarvis, 1998; Streit, Siegwolf, Hagedorn, Schaub, & Buchmann, 2014; Tissue, Thomas, & Strain, 1997; Yang et al., 2016). This fertilizer effect of CO_2_ has been attributed to an increase in the carboxylase activity and a reduction in the oxygenase activity of Rubisco due to the greater relative proportion of CO_2_ to O_2_ within chloroplasts (Ainsworth & Rogers, 2007; Rodrigues et al., 2016). As a result, increased [CO_2_] tends to significantly reduce photorespiratory metabolism (Drake, Gonzàlez‐Meler, & Long, 1997), which helps to explain the increase in growth rates observed in certain species. This increased [CO_2_] at Rubisco carboxylation sites also allows higher rates of *A* to be achieved at lower *g*_s_, resulting in reduced consumption of water per molecule of carbon fixed and therefore higher water use efficiency (WUE; Faralli et al., 2017; Franks, 2013; van der Sleen et al., 2014; Streit et al., 2014) (Figure 3). Together, the reduction in photorespiratory activity and the increase in WUE, induced by higher [CO_2_], have the potential to minimize the deleterious effects of certain abiotic stresses. In fact, some studies have demonstrated the mitigating effect of high [CO_2_] on plants exposed to drought and high temperatures (Drake et al., 2011; Oliveira et al., 2016; Rodrigues et al., 2016; Roy et al., 2016; Swann et al., 2016; Yang et al., 2016; Yu, Yang, Jespersen, & Huang, 2014). In view of this increase in performance and productivity promoted by high [CO_2_], even in the presence of abiotic stresses, should we really be concerned about the dynamics of the distribution and survival of forest species under a climate change scenario? The answer to this question is extremely complex because the beneficial effects of high [CO_2_] found in some studies (Drake et al., 2011; Idso & Kimbal, 1997; Oliveira et al., 2016; Pérez‐Jiménez, Hernández‐Munuera, Piñero, López‐Ortega, & del Amor, 2018; Radoglou & Jarvis, 1990; Rodrigues et al., 2016; Roy et al., 2016; Swann et al., 2016; Yu et al., 2014) are in direct contrast to the results of several other reports (Calvo et al., 2017; Clark, Clark, & Oberbauer, 2010; Faralli et al., 2017; Feeley, Joseph Wright, Nur Supardi, Kassim, & Davies, 2007; Voelker et al., 2017).

Although high [CO_2_] has the potential to increase growth rates, this effect is not always observed (Duursma et al., 2016; Feeley et al., 2007; Klein et al., 2016; van der Sleen et al., 2014) or, in many cases, is only transient (Grulke, Riechers, Oechel, Hjelm, & Jaeger, 1990; Warren et al., 2015) (Figure 3). Indeed, downregulation of photosynthesis is a common response of C3 plants exposed to high [CO_2_] levels, and it has been attributed to the inability of sink organs to utilize the excess photoassimilate produced (Drake et al., 1997; Makino & Mae, 1999; Rey & Jarvis, 1998). In turn, higher carbohydrate content in source organs may induce repression of several photosynthesis‐related genes, canceling the fertilizer effect of high [CO_2_] (Cheng, Moore, & Seemann, 1998; Nie, Hendrix, Webber, Kimball, & Long, 1995). Another factor commonly associated with the downregulation of photosynthesis is N availability, as N deficiency may limit the translocation capacity of source organs, as well as the growth and activity of sink organs (Ruiz‐Vera, Souza, Long, & Ort, 2017; Sharwood, Crous, Whitney, Ellsworth, & Ghannoum, 2017). In addition to N, some studies have demonstrated that P availability is another determining factor for the mitigating effect of high [CO_2_] under abiotic stress conditions, particularly drought (Jin, Lauricella, Armstrong, Sale, & Tang, 2015) (Figure 3). Nonetheless, P deficiency may offset the beneficial effects of exposure to high [CO_2_] and limit the productivity of forest species, even under conditions where increases in *A* are observed (Ellsworth et al., 2017).

Another point that is frequently debated is the potential of high [CO_2_] to mitigate the deleterious effects of certain abiotic stresses. Some studies have shown that the beneficial effects of high [CO_2_] can be offset by an increase in canopy leaf area (Becklin et al., 2017; McCarthy, Oren, Finzi, & Johnsen, 2006; Warren, Norby, Wullschleger, & Oren, 2011), reductions in root depth (Duursma et al., 2011), and alterations in xylem anatomical properties (e.g., increase in vessel diameter; Ceulemans, Jach, Velde, Lin, & Stevens, 2002). Such morpho‐anatomical changes induced by high [CO_2_] may result in increased transpiration demand and lower capacity to absorb and transport water due to reduced hydraulic conductivity of roots (Warren et al., 2011) and leaves (Domec et al., 2009) and higher vulnerability to cavitation in different organs (Domec, Schäfer, Oren, Kim, & McCarthy, 2010), exacerbating the susceptibility of plants to drought events. The same scenario of uncertainties about the mitigating effect of high [CO_2_] is also observed for plants exposed to high temperatures, particularly when this stress is associated with drought (Becklin et al., 2017; Duan et al., 2014). Under these conditions, increased leaf area and reductions in transpiration rates can significantly reduce latent heat loss, which may increase leaf temperature and subsequently generate a series of disturbances that may enhance vulnerability to heat stress (e.g., increase in *R* and *P*_R_ rates; Voelker et al., 2017) (Figure 3).

Finally, another matter of concern regarding plant behavior under a scenario of increased [CO_2_] is altered regulation of specific processes related to development, particularly phenology (Becklin et al., 2017). Several studies have shown that changes in flowering time appear to be a common response of plants exposed to high [CO_2_] (Jagadish et al., 2016; Springer, Orozco, Kelly, & Ward, 2008; Springer & Ward, 2007) (Figure 3). Such changes can have a devastating impact on the composition of several plant communities because changes in flowering time may, for instance, induce mismatches between plants and their respective pollinators and thereby compromise the reproduction and distribution of several species (Becklin et al., 2016; Polce et al., 2014; Springer & Ward, 2007). These results are even more worrying when we also consider the increase in global average temperature, another factor that has been directly associated with phenological changes in many species (Bock et al., 2014; Legave, Guédon, Malagi, Yaacoubi, & Bonhomme, 2015; Mulder, Iles, & Rockwell, 2017). Thus, it is clear that the effect of high [CO_2_] on the performance, survival, and distribution of species worldwide represents one of the greatest uncertainties related to climate change because its effects are extremely variable and also depend on a range of other factors.

## 4 IMPACT OF CLIMATE CHANGE ON SPECIES DISTRIBUTION AND FOREST COMPOSITION

**Figure 4:**
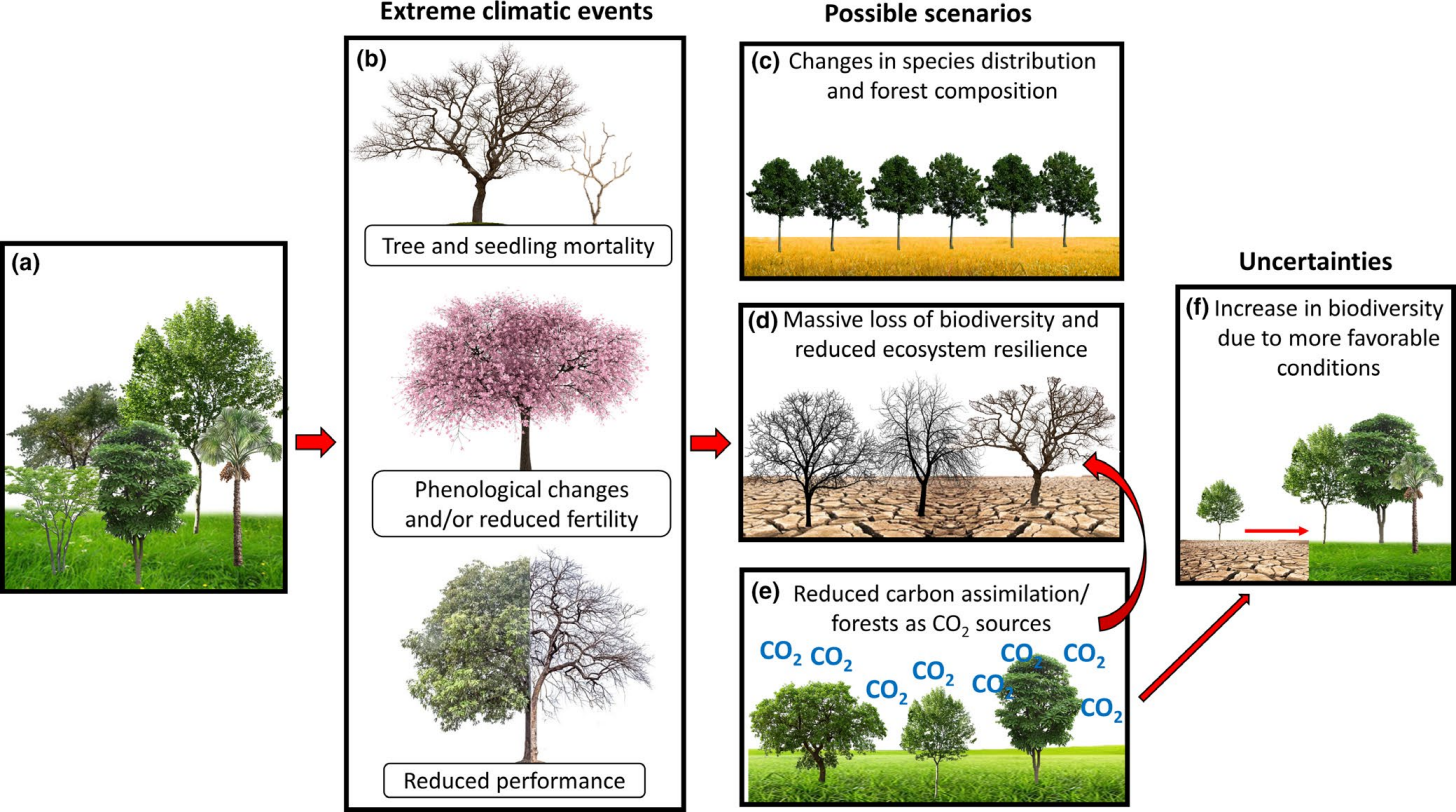
Climate change can induce profound transformations in forest ecosystems worldwide (a). The increase in frequency, intensity, and duration of extreme weather events can trigger massive tree mortality, affect species recruitment (due to alterations in germination, establishment, and early seedling survival), and reduce fertility and/or change the phenology of several species (b), resulting in deep changes in species distribution and forest composition (c). This massive loss of biodiversity can reduce the resilience of several forest ecosystems, making them even more sensitive to the effects of climate change (d). Even when the extreme weather does not induce tree mortality, the reduction in carbon assimilation and the increase in carbon release, due to reduced performance, can transform the forest ecosystem from carbon sinks to carbon sources, further increasing the atmospheric [CO_2_] (e), and thus the rate of climate change itself (e). This further increase in [CO_2_] can have contrasting effects, depending on the environmental context in which tree species are inserted. For several regions, the increase in [CO_2_] can enhance the deleterious effect of drought and high temperature (see also Figure 3), which may place species in a condition that exceeds their physiological limits, resulting in even more mortality events (d). Contradictorily, for other regions, the changes in temperature and precipitation patterns can lead to more favorable conditions to some species, which may result in an increase in performance and biodiversity on those regions (f)

As discussed above, the interaction between multiple climatic factors can trigger tree mortality due to the disruption of central physiological processes. Despite the uncertainties regarding the intensity, frequency, and duration of those extreme weather events for the decades to come (IPCC, 2014), even in more conservative scenario several tree species probably will be exposed to climatic conditions that differ significantly from their physiological limits (Becklin et al., 2016). Moreover, depending on the speed at which these stressors reach particular regions, the species inhabiting these regions may not have time to adapt to the new climatic conditions. Thus, if climate model predictions are confirmed, profound changes in the composition of several biomes can be expected (Wiens, 2016; Zhang et al., 2017). In fact, some studies on plant dynamics indicate that climate changes predicted for the end of this century could result in the replacement of current biomes by those that are more adapted (Jiang et al., 2013; Park Williams et al., 2012), including the replacement of the Amazon forest by savannah vegetation (Lapola, 2007), leading to huge losses of biodiversity (Figure 4).

In addition to the prediction from climatic models, changes in forest composition can be already seen in several regions around the globe. For example, a recent study had shown that the increase in drought intensity had led to a shift in species composition in forests of the eastern United States toward species that are more drought‐tolerant, but with lower growth rates (Zhang et al., 2018). Besides the losses in biodiversity, the resulting increase in mortality rates and reduction in forest biomass accumulation might also transform the global forest ecosystems from carbon sinks to carbon sources (Brienen et al., 2015; Cavaleri et al., 2017; Hisano, Searle, & Chen, 2018) (Figure 4c). This situation was already observed in forest ecosystems which are crucial to the regulation of carbon dynamics worldwide, like the Amazon forest (Brienen et al., 2015). Thus, it is clear that even small changes in forest composition can have a feedback effect which might increase the concentration of atmospheric CO_2_, and thus the rate of climate change itself (Phillips et al., 2009).

Alterations in forest composition can also result from shifts in the geographic distribution along climatic gradients (Hisano et al., 2018). In fact, several pieces of evidence support the latitudinal and altitudinal shifts induced by alteration in climatic conditions (Bellard, Bertelsmeier, Leadley, Thuiller, & Courchamp, 2012; Colwell et al., 2008; Hisano et al., 2018). As an example, expressive increments in atmospheric temperature, along with reductions in water availability, are attributed to the upward movement of trees from the lower elevation range boundaries and also to elevational range contractions in a forest from southeastern Arizona (Brusca et al., 2013). Similarly, an extreme drought event in the early 2000s leads to rapid vegetation redistribution in the southern California mountains (Fellows & Goulden, 2000). It is important to note that those shifts in plant distribution are highly species‐dependent since their environmental requirements and capacity to adapt are highly variable (Butler et al., 2017). Thus, in a given forest community, species that display a set of morpho‐physiological traits that confer higher tolerance to a given environmental will tend to increase their dominance, while more sensitive species tend to decay (Hisano et al., 2018; Moradi et al., 2012). This observation highlights the central role of biodiversity in minimizing the deleterious effects of climate change on forest communities (Hisano et al., 2018), since a more biodiverse system tends to be more resilient (Chapin et al., 2000; Grossiord, 2019; Sakschewski et al., 2016). This link between biodiversity and forest safety can be clearly observed in a recent study in which the diversity in hydraulic traits of trees was a central factor in mediating ecosystem resilience to drought (Anderegg et al., 2018).

Another concern regarding the changes in forest composition is related to tree recruitment since drastic changes in weather conditions can directly affect germination, establishment, and early seedling survival (Clark et al., 2016). In fact, some studies already showed a reduction in species richness due to reductions in seedling emergence and increased mortality following events of drought (Lucas‐Borja, 2016), especially when in association with high temperatures (Lloret & Pen, 2004; Lloret, Peñuelas, Prieto, Llorens, & Estiarte, 2009). This disruption in seedling recruitment might be further intensified by the direct effect of environmental variations on plant reproduction, both due to reductions in fecundity (Saavedra, Inouye, Price, & Harte, 2003; Su et al., 2013) and/or for mismatches between plants and their pollinators (as discussed above for high [CO_2_]) (Figure 4d,e).

Finally, it is important to highlight that, for some forest communities, the changes in climatic conditions can have a positive effect on plant biodiversity (Bellard et al., 2012; Hisano et al., 2018) (Figure 4f). For example, the increase in atmospheric temperature, in association with higher [CO_2_], can have a positive effect for many species (Rodrigues et al., 2016; Roy et al., 2016). Similarly, the increase in precipitation, predicted for some regions (IPCC, 2014), can also have a positive effect on threatened species, resulting in an increase in biomass production on those forest communities (Bellard et al., 2012). However, these results should be analyzed with care, since this controversial beneficial effect of climate change has been observed only on a small fraction of the vast literature that covers the impact of extreme weather events on plant function and composition. All these uncertainties add new layers of complexity to the already puzzling task of predicting the impact of climate change on the composition of forest communities worldwide.

## 5 CONCLUSIONS AND PERSPECTIVES

As discussed in the previous sections, there are multiple ways by which factors associated with climate change can increase the vulnerability of, as well as place at risk of extinction, numerous forest species distributed in the most diverse biomes around the world. Given this alarming scenario of potential changes in the composition of several plant communities, it is essential that more studies seek to elucidate the factors associated with climate change that may lead to plant mortality. Furthermore, more important than characterizing the factors related to plant vulnerability to a particular stress is determining how the interaction between multiple stressors can influence the survival of such species. In situations of exposure to multiple stressors, it is also of paramount importance to better characterize the real role of CO_2_, as well as the influence of variation in nutrient availability, in the mitigation or intensification of the deleterious effects of other stressors. In this sense, free‐air CO_2_ enrichment (FACE) and open‐top chamber (OTC) studies that simulate the concomitant occurrence of other stresses, such as drought and high temperature, are key to better characterizing the impact of climate change on plant behavior (Becklin et al., 2017). In addition, the use of integrative approaches from genomics, metabolomics, and proteomics, as well as those techniques used to monitor physiological changes in different organs (e.g., OV and micro‐CT methods), is of pivotal importance to trace a broader picture of the main limitations to plant performance under extreme weather events. The results obtained from such studies may provide valuable information for the optimization of models to monitor and predict the impact of climate change on the survival and distribution of plant species and public policies on forest management and reforestation.

## CONFLICT OF INTEREST

The authors declare that the research was conducted in the absence of any commercial or financial relationships that could be construed as a potential conflict of interest.

## AUTHOR CONTRIBUTION

All authors contributed equally to the design and writing of the manuscript.

## Data Availability

All data used in this study are included in the manuscript.
